# Book Reviews

**Published:** 1897-03

**Authors:** 


### Book Reviews

ARTIFICAL ANÆSTHESIA, a Manual of Anæsthetic Agents and their Employment in the Treatment of Disease, by Lawrence Turnbull, M. D., Ph. G., Aural Surgeon to the Jefferson Medical College Hospital, Philadelphia; late Honorary President of the Otological Snbsection of the British Medical Association, and of the Section of Laryngology and Otology of the American Medical Association. Fourth edition, revised and enlarged; 8 vo., cloth, pp. 550. Illustrated. Price $2.50. P. BLAKISTON, SON & Co., Publishers, 1012 Walnut Street, Philadelphia: 1896.

The fourth edition of this volume which was first issued in 1878, brings up the whole subject of artificial anæsthesia and anæsthetics to the present time, the latest local anæsthetic, eucaine being included. The modes of administering and chemical composition, of the several general and local anæsthetics, with precautions as to their use and their physiological effects follow two chapters of history and general consideration of the subject. The author warns against entrusting anæsthesia to inexperienced hands and advises that no one be allowed to administer any anæsthetic without a certain amount of tuition and no one should receive a diploma until he has shown a knowledge of the chemical composition and physiological action of such anæsthetics. Sound advice in view of the many deplorable accidents which could have been avoided.

The author gives preference to ether, and refers to it as "the one systemic anæsthetic which has proved, during all these years, the most available and most free from danger." The book is a very readable as well as valuable treatise.

THE DISEASES OF INFANCY AND CHILDHOOD.—For the use of Students and Practitioners of Medicine, by L. Emmet Holt, A. M., M. D., Prof. of Diseases of Children in the New York Polyclinic, etc., with two hundred and four illustrations, including seven colored plates. New York. D. Appleton & Co., 1897.

The recent substantial additions to systematic works in this province would seem to satisfy the demand, yet the excellence of this latest text-book will amply warrant its appearance and will without doubt rank it among the foremost treatises of its class.

The volume has been carefully prepared and shows evidence of painstaking research, large experience, and accurate observation on the part of its author. A unique feature of the book consists in the valuable object-lesson given in a series of photographs of the stomach from birth to eighteen months of age. If the actual capacity of the stomach was more generally known and considered in arranging the quantity of food given, infant mortality from overfeeding would be measurably decreased.

Mechanically the book is all that could be desired.

SWEDISH MOVEMENT OR MEDICAL GYMNASTICS, by Dr. T. J. Hartelins, Knight of the North Star Order, Knight of St.Olaf's Order, Director of the Central Gymnastic Institute of Stockholm, Sweden. Translated by A. B. Olsen, M. D., with introduction and notes by J. H. Kellogg, M. D., Superintendent Battle Creek (Mich.) Sanitarium and Hospital. Published by the Modern Medicine Pub., Co., Battle Creek, Mich. 1896.

This translation, the original of which is by the leading authority on Swedish movement, Hartelins, is a complete manual of the subject. It consists of two parts; the first dealing with positions and movements, and the second considering disease and its treatment by gymnastics. The list of diseases is a long one, embracing affections of the important organs as well as general morbid conditions, explicit directions for manipulation are given in every instance, and the meaning further elucidated by appropriate illustrations. Though very brief and concise, the whole subject is included in the 154 pages of text.

TWENTIETH CENTURY PRACTICE. An International Encyclopedia of Modern Medical Science. By leading authorities of Europe and America. Edited by Thomas L. Steedman, M. D., New York City. In Twenty Volumes. Volume VII. "Diseases of the Respiratory Organs and Blood, and Functional Sexual Disorders." New York: William Wood and Company. 1896.

The contributors to the seventh volume of this magnificent series are C. W. Allen, Comby of Paris, Cumston, Cushing, James M. French, E. Fletcher Ingals, E. Maier, of Paris; Riegel, of the University of Giessen; Alfred Stengel, Herbert B. Whitney. The subjects considered are: Diseases of the Pleura, Asthma, Hay Fever, Diseases of the Mediastinum, Diseases of the Diaphragm, Diseases of the Blood—Rachitis, Disorders of Menstruation, Functional Disorders of the Male Sexual Organs; Chemical and Microscopical Examination of the Urine. Each one of these articles is a complete treatise.

OVER THE HOOKAH, THE TALES OF A TALKATIVE DOCTOR, by G. Frank Lydston, M. D., Professor of Genito-Urinary Surgery in the Chicago College of Physicians and surgeons. Professor of Criminal Anthropology in the Kent College of Law, etc.

Sold by subscription only. Sent prepaid on receipt of subscription price. Price in cloth, gilt top, $4.00. Price in morocco, full gilt, $5.00. Over 600 pages, octavo. Profusely illustrated fron the author's designs, by C. Everett Johnson. The Fred Kline Publishing Co., Chicago.

